# Simulation in a high stakes clinical performance exam

**DOI:** 10.4103/0974-2700.50741

**Published:** 2009

**Authors:** Brad Peckler, Dawn Schocken, Richard Paula

**Affiliations:** Division of Emergency Medicine, Department of Medicine, College of Medicine, University of South Florida, Tampa General Hospital, 1 Davis Blvd Suite 504, Tampa, FL 33606, USA; 1 Division of Emergency Medicine, Department of Clinical Skills Lab, College of Medicine, University of South Florida, Tampa General Hospital, 1 Davis Blvd Suite 504, Tampa, FL 33606, USA

**Keywords:** Education, simulation, testing

## Abstract

**Background:**

Hi-fidelity simulation is becoming accepted as a teaching tool for medical providers. Advanced simulations allow educators to test difficult clinical scenarios. The goal of this study was to test the diagnostic and treatment skills of a third-year medical student faced with a simulated patient having evidence of a stable pneumothorax. Students are then expected to evaluate the teaching simulation in comparison to traditional methods.

**Methods:**

The case was one of a 12 cases in the “high stakes” Clinical Performance Exam. The patient with evidence of a stable pneumothorax was chosen to evaluate both diagnostic abilities and decision making in therapeutic options. Students were assessed using a university-wide standardized checklist: diagnosis, management, and interaction with the simulator. Immediately following the simulation, the students evaluated the experience.

**Results:**

The exam was given to 117 students. The correct diagnosis was made by 115/117 (98%). Treatment was considered acceptable in a majority of students, Send patient to the Emergency Department 77%, Oxygen 26% and Analgesia 39%. The follow-up survey completed by 78% of the students revealed the students felt comfortable with the simulators, but had concerns about the exam. Students liked the simulator as an educational tool 88% of the time.

**Conclusions:**

Simulation was used in a year-end exam and majority of students chose the correct diagnosis and treatment plan. It was also found that a significant percentage of students performed an unnecessary and potentially harmful procedure. The survey revealed that students were concerned about distractions and realism, but overall expressed desire for more education using simulation.

## INTRODUCTION

Hi-fidelity simulation is rapidly becoming accepted as an advanced teaching tool for medical providers in all stages of practice. Simulation can be used to demonstrate physical findings that would not be possible by other means.[1,2] Traditionally, trained patient-actors are used in skills labs to evaluate medical students' ability to perform a physical exam and assess their ability to interact with patients. The paid patients feign physical exam findings, and when necessary, the students are provided with cards or told of specific findings. This process becomes less effective when attempting to test whether specific physical findings can be identified by the student, and acted on appropriately. Simulation was used during a year-end "high stakes" Clinical Performance Exam (CPX). Simulation provides superior accuracy in determining student diagnostic capabilities than traditional card-based physical finding descriptions.[3–5]

The primary goal of this study was to test the diagnostic and treatment skills of a third-year medical student faced with a simulated patient having evidence of a stable pneumothorax. A secondary goal was to record student opinion on using a medical training simulation in the end of year CPX exam.

## MATERIALS AND METHODS

This observational study was approved by the Medical Dean of Students at the University of South Florida. A post hoc Institutional Review Board approval was obtained from The University of South Florida. A convenience sample of 117 third-year medical students taking their third-year final comprehensive CPX exam participated in the study. The exam consists of 12 clinical cases, all modeled after the United States Medical License Examination exam. The exam is required of all third-year medical students continuing into the fourth year. The exam was conducted over 12 nonconsecutive days within a one-month period. Students signed an honor code and were tested in groups of 10. A Medical Education Technologies Inc. (METI^®^) “Stan” hi-fidelity simulator was used.

The simulated scenario was a 47-year-old male, long-term smoker who presents to an outpatient clinic with pleuritic chest pain and shortness of breath. Symptoms began suddenly 3 weeks prior and have been persistent despite two prior clinic visits and two courses of antibiotics. A chest x-ray was not performed on either of the first two visits. The patient is stable according to vital signs and symptoms. The simulator has obvious decreased breath sounds on the affected side. After the history and physical exam the student may order a CXR, which shows a large, simple pneumothorax. The simulator interacts with the student in real time through a trained standardized patient (SP) actor using a wireless microphone observing behind one-way glass. The SP was trained by the center staff using a pre-determined script and was the same for all 12 exam days. The evaluation measured how students interpret physical findings, make a diagnosis, and formulate a treatment plan. The student was expected to make the diagnosis of pneumothorax.

Treatment included sending the patient to the Emergency Department for a chest tube or observation with oxygen. Correct medical treatments included oxygen, beta-2 agonists, and analgesia. Common incorrect treatment responses included needle decompression and antibiotics. The results were tallied using a computerized tallying system by B-Line Medical^®^. Students were assessed on their assessment, diagnosis, and management plan. The SP evaluated their interaction with the student by completing a case specific questionnaire. Two months after the exam, the students had a required visit to the center and were asked to evaluate their experience with this and other simulated CPX cases.

## RESULTS

The exam was given to 117 third-year medical students. The correct diagnosis was made by 115/117 (98%) students. The patient was sent to the emergency department for placement of a thorocostomy tube by 77% of the students. One student was given credit for admission to the hospital for observation on oxygen. Surgical treatment varied; 23% considered or preformed a needle decompression (incorrect) of the chest. Medical treatment also varied; oxygen (correct) was given by 26% of students; analgesia (correct) 39%; smoking cessation drugs (incorrect in the acute setting) 21%; antibiotics (incorrect) 8%; and miscellaneous incorrect medications and treatments 9%. The SP who played the voice of the patient felt that 89% of students displayed confidence in the ability to manage the case. During the physical exam, 95% of students auscultated the lungs properly.

**Figure 1:**
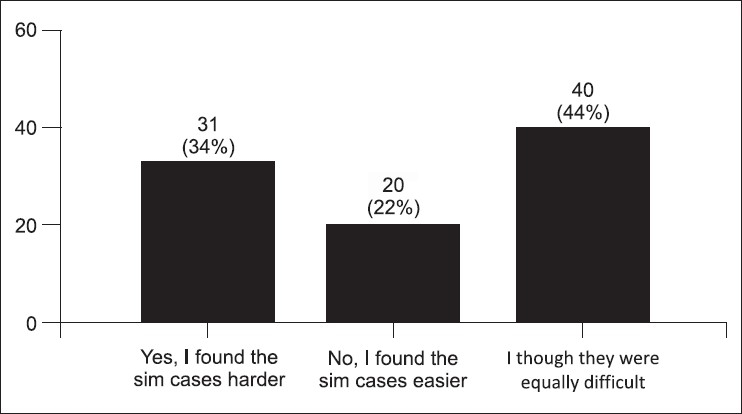
Were the simulator cases more difficult as compared to standardized patient cases? (91 responses)

**Figure 2:**
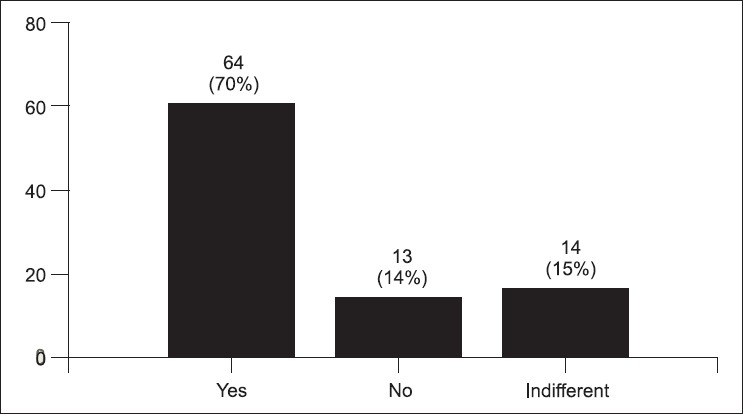
Did you find taking to a mannequin difficult? (91 responses)

**Figure 3:**
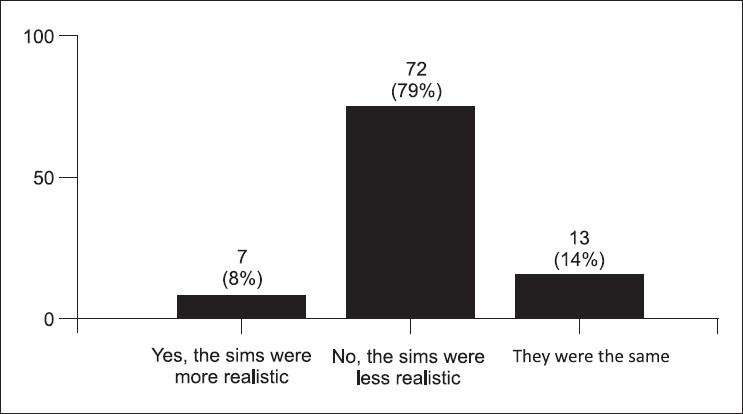
Was the simulator more realistic than a standardized patient? (91 responses)

**Figure 4:**
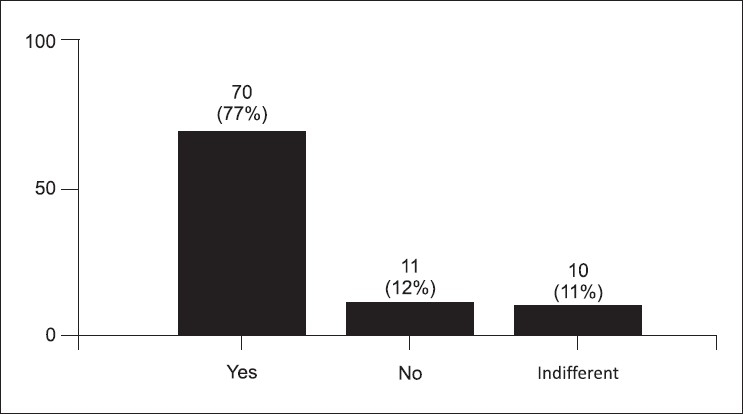
Would you like to have more teaching using the simulators? (91 responses)

**Figure 5:**
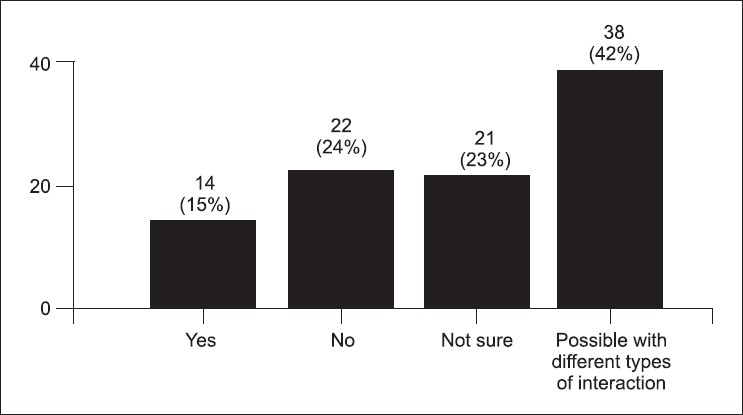
Would you like to see more tesing using the simulators? (91 responses)

The post-test survey was taken by 91 (78%) students. Some students stated that the simulator cases were more difficult (34%) [Figure 1]. Figures 2 and 3 demonstrate the students' concern about communication and realism. Also, 69% thought the simulator was a distraction. A third of the students described being uncomfortable and having fear of the unknown when using the simulator. Only 58% felt confident using the simulator in an exam, yet 88% felt confident using the simulator as an educational tool. Figures 4 and 5 describe some of the student's responses to questions about simulation in teaching and testing.

## DISCUSSION

The majority of the students made the correct diagnosis. The majority were also able to formulate the proper treatment plan. Interestingly, the subtleties of management of this case were lost on the most of our students. Oxygen, analgesia ,and beta-2 agonists for the respiratory conditions were only given by a third of the students. The different responses may be in part due the varied interests of specialties students will eventually enter. Along the same line of reasoning, it is understandable that 23% considered doing needle decompression, demonstrating the student acknowledges that the air must be released from the pleural space, but the urgency of the situation is misunderstood. The case was a simple stable pneumothorax without indication for emergent interventions. These results provided our instructors with specific areas of clinical competence on which to concentrate. The positive response from the SP regarding the patient interactions is commensurate with our traditional CPX cases.

The survey study revealed that the students did not feel comfortable with the simulators and were distracted by them. They remarked the simulators offered less realism and that it was difficult to communicate with them. Not surprisingly, few students wanted future simulation examination but 38% [Figure 5] were open to future simulation based exams. This is in concert with the fact that students did report a desire for additional educational opportunities to learn skills on hi-fidelity simulation. The survey reveals what has been shown previously in the literature; students like using the simulator for learning, but testing may be problematic.[6]

### Limitations

**Figure 6:**
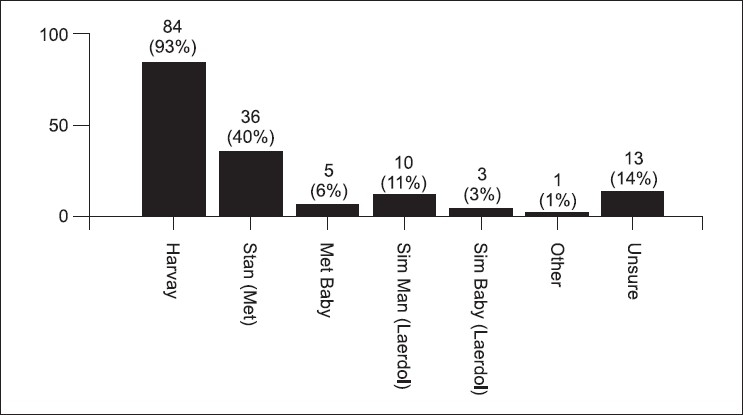
Have you ever worked on a hi-fidelity simulator prior to this exam? (90 responses)

There are several limitations to this study. This was the first CPX exam given using high fidelity simulators, and the data is from one medical school class. All members of the class have had some exposure to hi-fidelity simulation at least once during a mandatory Year Three Emergency Medicine rotation [Figure 6], but not necessarily the same type of simulator used in this study. The simulation program at this institution has only been recently created, and frequent simulation exposure is likely to have an effect on the experience.

## CONCLUSIONS

This paper describes the introduction of simulation in a high stakes medical school end of year CPX exam. Although the students expressed concerns with the simulated cases, most scored well. In the reported case, the majority of students identified the correct diagnosis and treatment, although a considerable number of students performed a potentially harmful procedure. Appropriate medical treatment was applied in only a third of the cases. This specific case would have been less powerful if done with a standardized patient. The physical findings would have been presented to the student in some fashion other than direct physical exam, preventing the ability to test student interpretations of actual physical findings. We believe these results are consistent with the diagnostic abilities of a third-year medical student. The survey results were contradictory in that the participants did not like the use of the simulator for the exam and had concerns about being tested on it, but did acknowledge that they like learning on it and the educational value of the experience. Further research could explore if using this technology more in the teaching process will make using simulators more acceptable to students in exam situations. Follow-up research will involve more study to validate hi-fidelity simulation as a testing tool.
